# The Effects of the Recombinant CCR5 T4 Lysozyme Fusion Protein on HIV-1 Infection

**DOI:** 10.1371/journal.pone.0131894

**Published:** 2015-07-08

**Authors:** Qingwen Jin, Hong Chen, Xingxia Wang, Liandong Zhao, Qingchen Xu, Huijuan Wang, Guanyu Li, Xiaofan Yang, Hongming Ma, Haoquan Wu, Xiaohui Ji

**Affiliations:** 1 Department of Microbiology and Immunology, Nanjing Medical University, 140 Hanzhong Road, Nanjing, Jiangsu Province, China; 2 Department of Neurology, The People’s Hospital of Jiangsu Province, 300 Guangzhou Road, Nanjing, Jiangsu Province, China; 3 Department of Neurology, The Second Hospital of Huaian, 62 Huaihai South Road, Huaian, Jiangsu Province, China; 4 Department of Neurology, Nanjing First Hospital, 68 Changle Road, Nanjing, Jiangsu Province, China; 5 Department of Neurology, Mingji Hospital of Nanjing, Jiangsu Province, 71 Riverside West Road, Nanjing, Jiangsu Province, China; 6 Department of Biomedical Sciences, Texas Tech University Health Sciences Center, 5001 El Paso Drive, El Paso, Texas, United States of America; Temple University School of Medicine, UNITED STATES

## Abstract

**Background:**

Insertion of T4 lysozyme (T4L) into the GPCR successfully enhanced GPCR protein stability and solubilization. However, the biological functions of the recombinant GPCR protein have not been analyzed.

**Methods:**

We engineered the CCR5-T4L mutant and expressed and purified the soluble recombinant protein using an *E*.*coli* expression system. The antiviral effects of this recombinant protein in THP-1 cell lines, primary human macrophages, and PBMCs from different donors were investigated. We also explored the possible mechanisms underlying the observed antiviral effects.

**Results:**

Our data showed the biphasic inhibitory and promotion effects of different concentrations of soluble recombinant CCR5-T4L protein on R5 tropic human immunodeficiency virus-1 (HIV-1) infection in THP-1 cell lines, human macrophages, and PBMCs from clinical isolates. We demonstrated that soluble recombinant CCR5-T4L acts as a HIV-1 co-receptor, interacts with wild type CCR5, down-regulates the surface CCR5 expression in human macrophages, and interacts with CCL5 to inhibit macrophage migration. Using binding assays, we further determined that recombinant CCR5-T4L and [^125^I]-CCL5 compete for the same binding site on wild type CCR5.

**Conclusions:**

Our results suggest that recombinant CCR5-T4L protein marginally promotes HIV-1 infection at low concentrations and markedly inhibits infection at higher concentrations. This recombinant protein may be helpful in the future development of anti-HIV-1 therapeutic agents.

## Introduction

CC chemokine receptor 5 (CCR5) belongs to the G protein-coupled receptor (GPCR) protein family. These proteins contain 7-transmembrane domains and mediate signal transduction events through their interaction with G proteins. CCR5 is a functional receptor for Chemokine (C-C motif) ligand 3 (CCL3 or MIP-1α), CCL4 (MIP-1β), CCL5 (RANTES), monocyte chemotactic protein (MCP)-2, and MCP-4 [1, 2]. It has been shown to be involved in the regulation of immune cell trafficking in a growing number of inflammatory diseases, such as rheumatoid arthritis, multiple sclerosis, and asthma [3,4], and acts as a crucial co-receptor for human immunodeficiency virus-1 (HIV-1) [5,6,7]. Individuals with mutant CCR5 are relatively resistant to HIV-1 infection and do not show apparent health problems [8, 9, 10], indicating that CCR5 is an ideal target for treatment and prevention of HIV-1 infection. The first CCR5-blocking drug, maraviroc, was approved in 2007 [11, 12].

GPCRs form the largest superfamily of drug targets. Therefore, their three-dimensional structural and dynamic information is of great interest to researchers so that effective drugs that target GPCRs can be designed [13]. Several CCR5 structural models have been reported in the literature [14–18], most of them homology models built on a bovine rhodopsin structural template. More recently, a three-dimensional structure of CCR5 bound to the HIV-1 drug maraviroc was solved [19]. However, this is only a single snapshot of the structurally diverse CCR5 molecule. The initiation and subsequent termination of HIV infection still remains an enigma.

The insertion of T4 lysozyme (T4L) into intracellular or extracellular receptor loops has contributed to most recently available structures of GPCRs. The creation of T4 lysozyme fusions has facilitated the structural determination of β2 adrenergic [20–21], A2a adenosine [22], dopamine D3 [23], chemokine CXCR4 [24], histamine H1 [25], lyso-phospholipid S1P [26], M2/ M3 muscarinic acetylcholine [27–28], and δ/κ/μ-opioid receptors [29–31]. All reported that functional analyses of T4L-GPCR fusions are limited to ligand binding; assays of chemokine receptor function and modulation of HIV-1 co-receptor activities (CXCR4 and CCR5) by T4L-GPCR fusion constructs have not been reported [19,24]. Therefore, detailed knowledge of CCR5 structure and dynamics is critical in understanding its functions and/or dysfunctions in the rational design of selective therapeutic compounds. Such studies would require the reliable production of functional CCR5 on the tens of milligram scale.

Here we report that a T4 lysozyme fusion CCR5 variant protein (CCR5-T4L) was purified as a soluble recombinant protein (in milligram amounts) using a pET20b expressing system. We investigated the effects of soluble CCR5-T4L on viral infection in cell lines, primary human macrophages, and PBMCs from different donors. We demonstrated that soluble recombinant CCR5-T4L protein acts as HIV-1 co-receptor, interacts with wild type CCR5, down-regulates surface CCR5 expression in human macrophages, and interacts with CCL5 to inhibit CCL5-induced macrophage migration. We examined the different binding properties of CCR5-T4L and wild type CCR5 using [^125^I]-CCL5 and [^35^S]-GTPγS binding assays. The results of this study may be useful for the future design and development of anti-HIV-1 therapeutic agents.

## Materials and Methods

### Cells and other reagents

The ethics committee of Nanjing Medical University approved our research plan. All study participants provided written informed consent. The ethics committee approved the consent procedure. HeLa, HEK-293, and THP1 cell lines were obtained from the American Type Culture Collection (Rockville, MD). 3T3.T4 cells were obtained from the NIH AIDS Reagent Program. All cell lines were maintained in DMEM (Quality Biologicals, Gaithersburg, MD) containing 10% (vol/vol) fetal bovine serum (FBS), 2 mM glutamine, and 2 mM penicillin-streptomycin. Phycoerythrin (PE)-conjugated 2D7 and PE-conjugated 3A9 monoclonal antibodies were purchased from BD Biosciences (San Jose, CA). Mouse monoclonal (sc-32304) and goat polyclonal (sc-6128) anti-CCR5 antibodies were purchased from Santa Cruz Biotechnology, Inc. (Dallas, TX). The mouse monoclonal His-tag antibody was purchased from Abcam (Cambridge, MA). [^35^S]-GTPγS was from GE Healthcare Bio-Sciences (Piscataway, NJ). Plasmids encoding T4 lysozyme WT were purchased from Addgene (Cambridge, MA). TAK-779, maraviroc, and the plasmids PcREV and pNL4-3.Luc+env− were obtained from the NIH AIDS Reagent Program. The PET-20b expression vector and BL21-Gold (DE3) pLysS *E*. *coli* strain were purchased from Stratagene (La Jolla, CA). Ni-nitrilotriacetic acid (NTA) histidine binding beads were purchased from Sigma-Aldrich (St. Louis, MO). All detergents were purchased from Affymetrix (Santa Clara, CA). All other reagents unless indicated were from Sigma-Aldrich. Soluble CD4 and chemokine CCL5 were purchased from R&D Systems (Minneapolis, MN).

Laboratory-adapted HIV-1 strains IIIB and BaL as well as primary R5 HIV-1 isolates (92US657, 93MW959 and 92TH009) were obtained from the NIH AIDS Research and Reference Reagent Program. In addition, recombinant HIV-1 gp160 proteins from HIV-1 isolates SF162 and BaL were obtained from the NIH AIDS Research and Reference Reagent Program.

### Generation of T4L-fused CCR5 constructs

Human CCR5 in the pSC-59 vector and T4 lysozyme WT vector were used as the starting templates for generating T4L-fused CCR5 constructs. PCR was performed using the FastStart High Fidelity PCR System (Roche, Shanghai, China). The amplification protocol consisted of 5 min denaturation at 94°C, followed by 30 cycles of denaturation at 94°C for 1 min, annealing at 55°C for 1 min, and 72°C for 1 min. Amplified DNA was purified from agarose gels using the PureLink Quick Gel extraction kit (Invitrogen, Shanghai, China). The entire CCR5-T4L DNA fragment without a stop codon was further subcloned into the pET-20b expression vector using the restriction enzyme sites BamHI and XhoI, whereas the CCR5-T4L fragment with a stop codon was cloned into pSC59-IRES-EGFP vector using the restriction enzyme sites NheI and SacII. Both T4L-fused CCR5 constructs were confirmed by DNA sequencing (Genomics Core Laboratory, Texas Tech University Health Sciences Center, El Paso, TX).

### Expression and purification of recombinant His-tagged T4L fused CCR5 constructs

We transfected the recombinant pET-20b/ CCR5-T4L vector into gold BL21(DE3) pLysS *E*. *coli* as previously described [32]. Briefly, a single colony was inoculated into 5 ml LB broth containing ampicillin (100 μg/ml) and chloramphenicol (34 μg/ml). Cultures were grown overnight at 37°C with shaking. After approximately 16 h, inoculations were added to 1 L LB broth containing ampicillin (100 μg/ml) and chloramphenicol (34 μg/ml). Cultures were grown at 37°C at 200 rpm until an OD_600_ of 2.6 was achieved. CCR5-T4L expression was induced with a final concentration of 1 mM isopropyl β-D-thiogalacto-pyranoside (IPTG), and cultures were shaken for 48 h at 125 rpm at 20°C. Supernatants were discarded, and pellets were air dried and stored at -80°C until purification.

Cell pellets were suspended in 5 ml lysis buffer (50 mM sodium phosphate [pH 7.8], 200 mM NaCl, 100 mM KCl, 20% glycerol, 10 mM EDTA, 2 mM DTT, 1 mM PMSF, 50 μg/ml lysozyme, 20 μg/ml DNase I) per 1 g of pellet followed by 3 freeze-thaw cycles on dry ice and 37°C. Cells were lysed by passing through a French press at 18,000 psi. Cell debris was removed by centrifugation at 6,600 g for 15 min. The supernatant was centrifuged at 126,000 g for 1 h to collect the membrane fraction and soluble fraction. The soluble fraction was added to a 5 ml-bed-volume Ni-nitrilotriacetic acid (NTA) histidine-binding column (Sigma, St. Louis, MO) equilibrated with 10 column volumes of binding buffer (50 mM sodium phosphate [pH 7.8], 200 mM NaCl, 100 mM KCl, 20% glycerol, 10 mM EDTA, 2 mM DTT, 1 mM PMSF, 50 μg/ml lysozyme, 20 μg/ml DNase I, and 1 tablet Roche Complete protease inhibitors in 50 ml buffer). Columns were washed with increasing concentrations of imidazole to remove non-specific bacteria contaminants and eluted with 5 ml elution buffer (20 mM sodium phosphate, 0.5 M NaCl, 0.5 M imidazole [pH 7.3]).

The membrane fraction was suspended in 5 ml membrane lysis buffer (20 mM sodium phosphate, 0.5 M NaCl [pH 7.8], 1% FC-12) per 1 g pellet and rotated for 2 h at room temperature. The clarified supernatant was purified using 5 ml-bed-volume NTA histidine column-binding beads equilibrated with 10 column volumes of binding buffer. The column was rotated overnight at 4°C and washed consecutively with 10 ml of 20 mM sodium phosphate, 0.5 M NaCl (pH 7.8), 0.05% FC-12, and 20 and 40 mM imidazole to remove non-specific bacterial contaminants. Proteins were eluted with 3 ml elution buffer (20 mM sodium phosphate, 0.5 M NaCl, 0.5 M imidazole [pH 7.3], and 1% FC-12). The large amount of soluble or membrane fraction was purified by fast protein liquid chromatography(FPLC) using a AKTA purifier(GMI, Inc, Ramsey, MN). The elution buffer was removed from purified proteins using a PD-10 column equilibrated with phosphate-buffered saline (PBS) and 0.2% FC-12. Fractions (1 ml) were collected and measured at OD600. The peak fractions were pooled and quantified using Bradford assays (Bio-Rad Laboratories, Hercules, CA). The resulting purified recombinants were stored at -80°C.

### HIV-1 envelope-mediated cell fusion assays and single-round virus infection assays

HIV-1 env-mediated fusion was assayed using a Vaccinia-based reporter assay as previously described [32]. Briefly, the target THP-1 cells were infected with Vaccinia virus encoding the bacteriophage T7 RNA polymerase. Effector HeLa cells were co-infected with PT7-lacZ Vaccinia virus and Vaccinia virus encoding either R5 BaL env or the control Unc (an uncleavable HIV-1 env that has a mutated cleavage site and therefore cannot engage in membrane fusion). The Unc Env is used to measure the non-specific background in the fusion assay. The target or effector cells were plated in 96-well plates (1×10^5^ cells) per well and treated for 1 h with or without either soluble recombinant CCR5-T4L or CCL5. After incubation for 1 h at 37°C and 6% CO_2_, Env-expressing effector cells were mixed with target cells (1:1 ratio). The cell mixtures were incubated for 2.5 h at 37°C, lysed, and the substrate chlorophenol red-β-D-galactopyranoside (CPRG) was added. The extent of cell fusion was assayed by measuring the amount of β-galactosidase produced.

We adapted the improved pseudotyping assay developed by He et al. (1995) to prepare pseudotyped virus particles. 293T cells were cotransfected using standard calcium phosphate (Promega, Madison, WI, USA) with 5 μg of PcREV and 20 μg of the envelope-defective luciferase-transducing HIV proviral clone pNL4-3.Luc+env− in the presence or absence of 10 μg of HIV-1 BaL—Env expression vectors. Fifteen hours after transfection, 20 mM sodium butyrate and fresh media were added. The supernatants were harvested after 48–72 h by centrifugation at 1500 rpm for 5 min and filtered through a 22-μm filter. Virus stocks were stored at -80°C. Vesicular stomatitis virus G glycoprotein (VSV-G) was used as a positive control to verify the competence of cells in cell fusion. HIV-1 p24 antigen was quantified by ELISA (ABL, Inc. Rockville, MD) and normalized to luciferase activity (relative luminescence units per minute) by titration of U373 cell viral supernatants. Pseudotyped virus infection assays were performed by incubating 3×10^5^ THP-1 cells (containing 5 μg/ml polybrene) infected with 1 ml of virus stock for 4–5 h at 37°C in a six-well plate. Following incubation, cells were washed with 10% FBS, fresh media were added, and the cells were incubated for another 48 h. The cells were harvested, and luciferase assays were performed using the luciferase assay system as recommended by the manufacturer (Promega, Madison, WI).

### Anti-HIV-1 assays with human PBMCs and monocytes/macrophages

Human PBMCs and monocytes were isolated from healthy donors by Ficoll-Hypaque gradient centrifugation. Assays for anti-HIV-1 activity were performed using 3-day-old phytohemagglutinin and interleukin-2-stimulated PBMCs or 6-day-old cultured monocytes/macrophages. All experiments for anti-HIV-1 activity were performed with triplicate samples in RPMI 1640 media supplemented with 15% heat-activated FBS, L-glutamine (2 mM), penicillin (100 U/ml), and streptomycin (100 μg/ml). HIV-1 replication in PBMC and monocytes/macrophage cultures was determined by measuring the RT activity, *gag* RNA, and p24 protein. Cell viability was determined by measuring formazan dye reduction in replicate cultures using Cell Titer reagent (Qcbio S&T, Shanghai, China). The HIV RT inhibitor AZT was used as a positive control for all assays.

### RNA extraction and quantitative RT-PCR

Total RNA from monocytes/macrophages, stimulated by M-CSF for 48 h, was isolated using TRIzol reagent (Life Technologies, Grand Island, New York). Total RNA (1 μg) was reverse transcribed using an RT system (Promega, Madison, WI, USA) with random primers for 1 h at 42°C. The reaction was terminated by incubating the reaction mixture for 5 min at 99°C. Mixtures were kept at 4°C. 0.1μg cDNA was then used as a template for real-time PCR quantification using iQ SYBR Green Supermix (Bio-Rad Laboratories, Hercules, CA, USA). The amplified products were visualized and analyzed using MyiQ software provided with the thermocycler (Bio-Rad Laboratories, Hercules, CA, USA). Oligonucleotide primers were synthesized by Integrated DNA Technologies (Coralville, IA, USA).

### Ligand binding assays and [^35^S]GTPγS binding assays

Chemokine binding assays were conducted as previously described [32]. Briefly, HEK-293 cells were harvested in PBS containing 0.5% bovine serum albumin and 0.01% sodium azide 48 h after transfection with T4L-fused CCR5 constructs. Specific binding was performed by incubating 1×10^6^ cells with 0.3 nmol/L [^125^I]-CCL5 (2000 Ci/mmol; PerkinElmer, Massachusetts). Non-specific binding was determined by the addition of 1 μM unlabeled CCR5-T4L or CCL5. After 2 h at 37°C, unbound chemokines were separated from cells by pelleting through a 10% sucrose/PBS cushion. Bound ligands were quantified by counting gamma emissions.

[^35^S]GTPγS binding assays were carried out as described [33]. Cells were lysed at 4°C in 5 mM Tris-HCl (pH 7.5), 5 mM EDTA, and 5 mM EGTA. After centrifugation at 30,000 g, the pellet was resuspended, and aliquots containing 10 μg protein were incubated at 30°C for 1 h in 50 mM Tris-HCl (pH 7.5), 1 mM EDTA, 5 mM MgCl_2_, 100 mM NaCl, 40 μM GDP, 0.5 nM [^35^S]GTPγS (1,200 Ci/mmol) in the presence or absence of the agonists. The reaction was terminated by adding cold PBS and filtered through GF/C filters, which were counted in a liquid scintillation spectrophotometer. Basal binding was determined in the absence of agonists, and nonspecific binding was obtained in the presence of 10 μM GTPγS. The percentage of stimulated [^35^S]GTPγS binding was calculated as 100 × (cpm_sample_−cpm_nonspecific_) / (cpm_basal_−cpm_nonspecific_).

### Chemotaxis assays

Chemotaxis assays were performed as previously described [32]. Briefly, for CCL5-mediated chemotaxis, macrophages were suspended in chemotaxis media (Iscove's modified Dulbecco's media (IMDM)) supplemented with 0.5% bovine serum albumin (BSA) at a density of 2×10^6^ cells/ml. The chemokine CCL5 (100 nM), with or without purified recombinant T4L-CCR5 protein (100 nM), was suspended in chemotaxis media. These mixtures were added to the wells of a 24-well plate followed by placement of a 5-micron pore transwell membrane (Costar, Corning Incorporated, Acton, MA). Cells were added on the transwell membrane at a volume of 0.1 ml (2×10^5^ cells). The plates were then capped and incubated for 4 h at 37°C in 5% CO_2_. The transwell membranes were then removed from the wells and the cells that migrated were counted using flow cytometry.

### Flow cytometry

HEK-293 cells were transfected with pSC-IRES-GFP-CCR5-T4L vectors using DOTAP (Roche Diagnostics, Indianapolis, IN) and then activated by Vaccinia virus infection. Fluorescent-activated cell sorting (GFP positive) was carried out 16 h after infection and the level of CCR5 expression was measured by flow cytometry using a phycoerythrin (PE)-conjugated 2D7 and a phycoerythrin (PE)-conjugated 3A9 monoclonal antibody. Briefly, after washing, cells were incubated with 1 μg /ml 2D7 or 3A9 at room temperature for 15 min. After washing again, cells were fixed in 1% paraformaldehyde/PBS. The red (CCR5) fluorescence was analyzed with a FACScan flow cytometer (Becton Dickinson, San Jose, CA, USA). Results were expressed as the percentage of PE positive cells in the GFP-positive fraction. For intracellular staining, a Cytofix/Cytoperm kit from BD Biosciences (San Jose, CA) was used. MDM was pretreated with or without soluble recombinant CCR5-T4L protein for 2 h at 37°C in 5% CO_2_. After the incubation period, the soluble recombinant protein was removed by washing at least three times using PBS and the cells were analyzed by flow cytometry.

### Gel electrophoresis and western blotting

Protein samples (15 μl) were mixed with the same volume of 2× SDS loading buffer and incubated at 37°C before loading on an SDS-PAGE gel. The transfer was performed at 100mA and 25 Volts constant current for 1 h in transfer buffer (1×Tris-Glycine buffer containing 20% methanol, Bio-Rad Laboratories, Hercules, CA). PVDF membranes were incubated with the mouse monoclonal His-tag antibody (1:2000 dilution) for 2 h, followed by three washes for 5 min with TBST buffer. Subsequently, membranes were incubated for 1 h with the goat anti-mouse IgG/M HRP antibody (1:5000 dilution). After washing four times (5 min each) with TBST buffer, blots were visualized using ECL plus western blot detection reagents (GE Healthcare Life Sciences, Shanghai, China). For co-immunoprecipitation experiments, cell products were prepared as previously described [34]. After pre-clearing cell extracts with protein G-agarose, samples were divided in half and immunoprecipitated with the CCR5 C-terminal antibody produced in goat (sc-6128) prebound to protein G beads. Wild type CCR5 (WT-CCR5) protein was precipitated with the CCR5 N-terminal antibody (sc-32304), and the CCR5-T4L protein was probed with the anti-6×His antibody.

### Statistical analyses

All experiments were performed at least three times, and the results of representative experiments are presented. Graphs were generated using GraphPad Prism 5. If the sample size was less than fives, we did not perform statistical analyses. All data are expressed as the mean ± standard deviation (SD).

## Results

### Recombinant CCR5-T4L acts as a HIV-1 co-receptor and down-regulates wild type CCR5 expression on the 3T3.T4 cell surface

**Fig 1:**
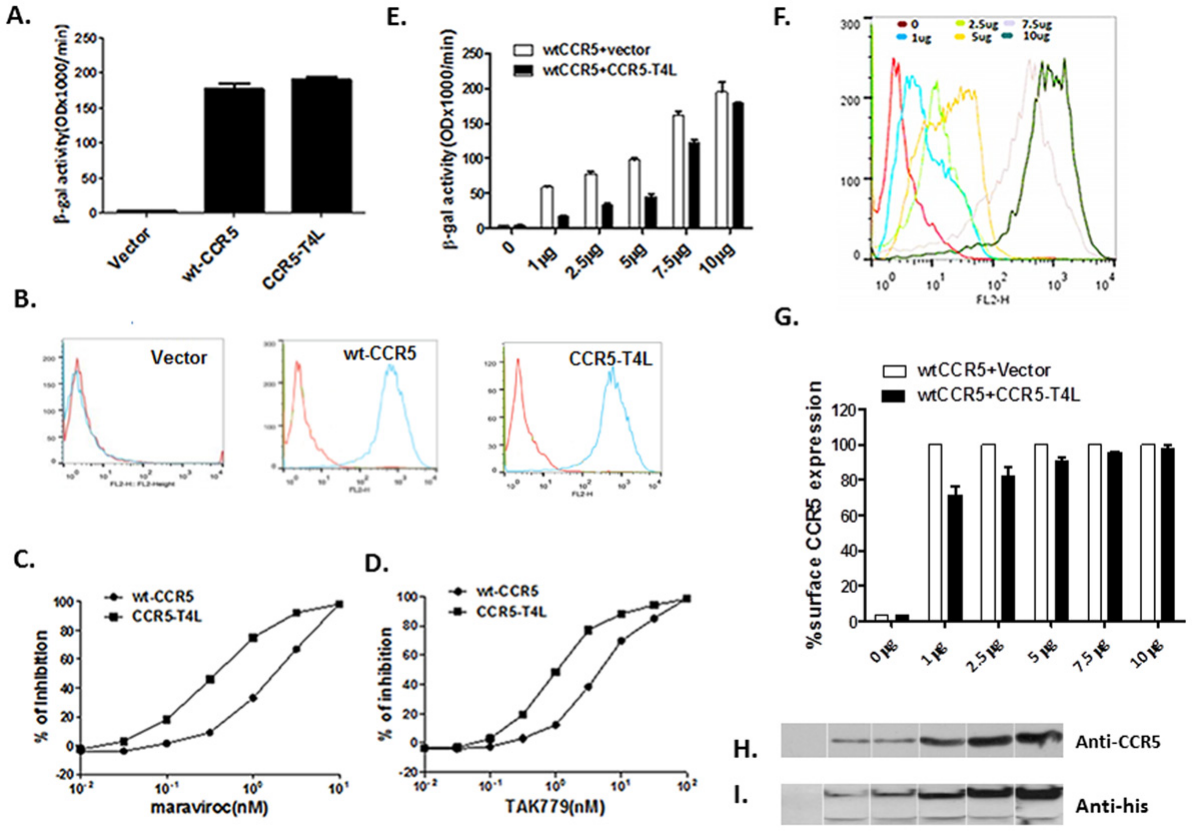
Recombinant CCR5-T4L acts as a HIV-1 co-receptor and down-regulates WT-CCR5 expression on the 3T3T4 cell surface. A) HIV-1 co-receptor function was estimated using cell-cell fusion assays. 3T3.T4 cells were transfected with 15 μg pSC-CCR5-T4L, then infected with vaccinia virus encoding LacZ gene under T7 promotor and used as target cells. The target cells were mixed with HeLa effector cells, expressing BaL Env protein and bacteriophage T7 RNA polymerase. After incubation for 2.5 h at 37°C, the amount of β-galactosidase activity was measured. WT-CCR5 was used as a positive control. B) CCR5 expression on the cell surface was confirmed using FACS. The level of CCR5 expression on above target 3T3.T4 cells was measured by flow cytometry using either phycoerythrin (PE)-conjugated 2D7 or phycoerythrin (PE)-conjugated 3A9 monoclonal antibodies. C and D) CCR5-T4L was more sensitive to CCR5 agonists maraviroc and TAK-779 inhibition. Different concentrations of maraviroc or TAK-779 were used to pretreat target 3T3.T4 cells transfected with pSC-CCR5-T4L or pSC-CCR5 for 1 h at 37°C. After washing with PBS, cell-cell fusion was performed. E and F) CCR5-T4L inhibited WT-CCR5 by reducing its expression on the cell surface. Different amounts of CCR5-T4L or WT-CCR5 cDNA (0 μg、 1 μg、 2.5 μg、5 μg、 7.5 μg and 10 μg) were co-transfected into 3T3.T4 cells. The effect of R5 HIV-1 Env-mediated cell-cell fusion was examined (E) and the level of CCR5 expression on the cell surface was measured by flow cytometry using a PE-conjugated monoclonal antibody (2D7) (F). G) The inhibition by CCR5-T4L decreased as the amounts of CCR5-T4L and WT-CCR5 increased. CCR5 expression was measured using flow cytometry and a PE-conjugated monoclonal antibody (2D7). The averaged mean fluorescence values (MFVs) for CCR5 from three experiments are plotted as a bar diagram, where WT-CCR5 expression after transfection with WT-CCR5 plus empty vector is set at 100. H and I) The interaction between CCR5-T4L and WT-CCR5 was tested using co-immunoprecipitation. 3T3.T4 cells co-transfected with CCR5-T4L and WT-CCR5 were lysed with RIPA buffer. Lysates were prepared and immunoprecipitated with the CCR5 C-terminal antibody, fractionated by SDS-PAGE, and immunoblotted. Blots were probed with the N-terminal CCR5 antibody (H), stripped, and reprobed with the anti-6×His antibody (I). Following the primary antibody reaction, blots were washed and probed with the anti-mouse antibody conjugated to HRP. Blots were exposed to X-ray film after reaction with the HRP substrate.

To assay the co-receptor function of CCR5-T4L, we transfected CCR5-T4L into 3T3.T4 cells and measured fusion efficiency using Env-mediated cell-cell fusion assays. Wild type CCR5 was used as a positive control. Expression of CCR5-T4L in 3T3.T4 cells resulted in efficient fusion of 3T3.T4 cells with HeLa-R5-BaL Env cells (Fig 1A), which can be detected on the cell surface using 2D7 and 3A9 monoclonal antibodies (Fig 1B). As shown in Fig 1B, both CCR5-T4L and WT-CCR5 were expressed on the 3T3.T4 cell surface. The results showed that CCR5-T4L has the same HIV co-receptor function as WT-CCR5. Furthermore, CCR5-T4L was more sensitive to the CCR5 agonists maraviroc and TAK-779 inhibition compared to WT-CCR5. As shown in Fig 1C and 1D, in CCR5-T4L expressing cells, IC_50_ values for maraviroc and TAK-779 were 0.6±0.23 nM and 1.67±0.49 nM, respectively. In contrast, wild type CCR5-expressing cells had IC_50_ values of 5.3±1.02 nM and 11.5±2.3 nM, respectively.

To further characterize the interaction between CCR5-T4L and WT-CCR5, we co-transfected different amounts of CCR5-T4L and WT-CCR5 into 3T3.T4 cells. We compared fusion efficiency using Env-mediated cell-cell fusion assays and evaluated WT-CCR5 cell surface expression using FACS. Although CCR5-T4L has the same HIV co-receptor function as WT-CCR5, we found that CCR5-T4L could reduce the fusion signal induced by WT-CCR5. As shown in Fig 1E, CCR5-T4L dominantly inhibited R5 Env-mediated cell-cell fusion signal when CCR5-T4L and WT-CCR5 (1 μg each) were co-transfected into 3T3.T4 cells. This inhibition decreased as the amounts of CCR5-T4L and WT-CCR5 increased. As shown in Fig 1F, cell surface CCR5 expression increased after co-transfected cells with more CCR5-T4L and WT-CCR5 plasmid DNA. Fusion levels depended on the amount of CCR5 expressed on the surface of 3T3.T4 cells (Fig 1E and 1G).

We further examined the interaction between CCR5-T4L and WT-CCR5 using co-immunoprecipitation experiments. After co-immunoprecipitating with the anti-C-terminal CCR5 antibody, samples were probed with the N-terminal CCR5 antibody, resulting in a 45-kDa WT-CCR5 protein band (Fig 1H). In contrast, approximately 85-kDa and 35-kDa protein bands, corresponding to the CCR5-T4L protein and T4L protein, respectively, were detected with the anti-His antibody (Fig 1I). Because the anti-C-terminal CCR5 antibody cannot detect CCR5-T4L, these results suggested that WT-CCR5 interacts with CCR5-T4L.

### Expression and purification of soluble recombinant CCR5-T4L protein in an *E*. *coli* system

**Fig 2:**
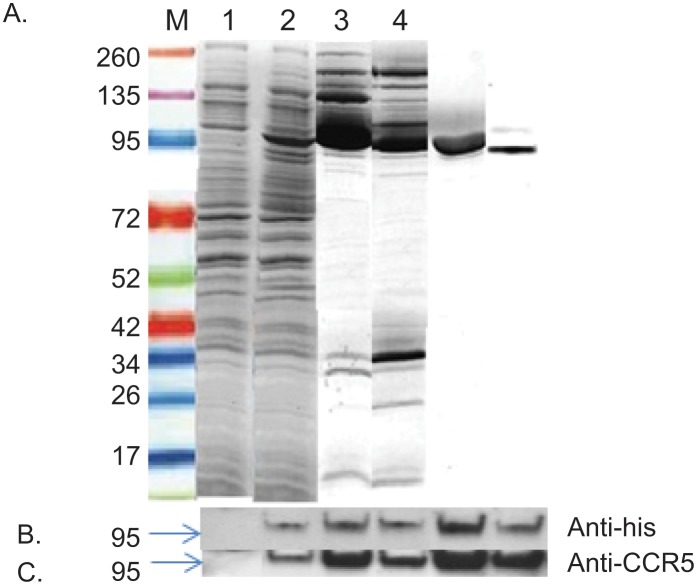
Expression and purification of soluble recombinant CCR5-T4L protein in an *E*. *coli* system. A) Large scale purification and identification of recombinant CCR5-T4L. Recombinant CCR5-T4L was expressed in *E*. *coli*. The pET-20b expression vector was transformed into Rosetta 2 (DE3) golden BL21 pLysS cells and analyzed using Coomassie brilliant blue R-250. Lane M: protein marker; lane 1: uninduced bacterial lysate; lane 2: IPTG-induced bacteria lysate; lane 3: small amount of soluble fraction purified on a Ni-nitrilotriacetic acid (NTA) histidine-binding column; lane 4: small amount of membrane fraction purified using a Ni-nitrilotriacetic acid (NTA) histidine-binding column; lane 5: large amount of soluble fraction purified by fast protein liquid chromatography (FPLC) using an AKTA purifier; lane 6: large amount of membrane fraction purified by fast protein liquid chromatography (FPLC) using an AKTA purifier. B and C) Western blot analyses using the anti-6×His tag monoclonal or anti-human CCR5 monoclonal antibodies (3A9).

Large-scale expression was successfully performed in a 5 liter shaking flask filled with 1 liter LB culture media. The expression of recombinant CCR5-T4L protein was induced by IPTG, and recombinant CCR5-T4L was induced with a final concentration 1 mM IPTG at 20°C for 48 h. Cell pellets were stable at -80°C for at least six months. In order to optimize high-level CCR5-T4L production, many variables were systematically studied, including timing, length of induction, and incubation temperature. The yield was significantly higher at 20°C than at 37°C. A further decrease of temperature to 15°C or a decrease of IPTG concentration from 1 mM to 0.4 mM resulted in lower yields. In addition, increasing cell density successfully maximized the yield. The highest recombinant protein expression was observed 48 h post induction at OD_600_ around 2.6. *E*. *coli* cell lysates were separated into the soluble cytoplasmic fraction (sol) and insoluble membrane fraction (mem). After a two-step purification from the soluble cytoplasmic fraction sample, we obtained soluble recombinant CCR5-T4L protein with a single visible band (~95% pure as estimated by SDS-PAGE). Soluble recombinant CCR5-T4L protein was found in both soluble and membrane fractions at the same molecular weight of approximately 85 kDa (Fig 2A). Western blot analyses with the anti-human CCR5 antibody (NT) and anti-His tag antibody (anti-6x his) confirmed the identity of the 85 kDa band (Fig 2B and 2C).

### The effects of CCR5-T4L protein on R5-tropic HIV-1 infection and replication in THP1 cells

*In vitro* assays, including cell-cell fusion assays, single-cycle viral infection assays, and replication-competent viral assays, were conducted to evaluate the antiviral potencies of soluble recombinant CCR5-T4L protein. Control solutions, including recombinant ligand CCL5 and PBS, were tested in parallel with soluble recombinant CCR5-T4L protein.

**Fig 3:**
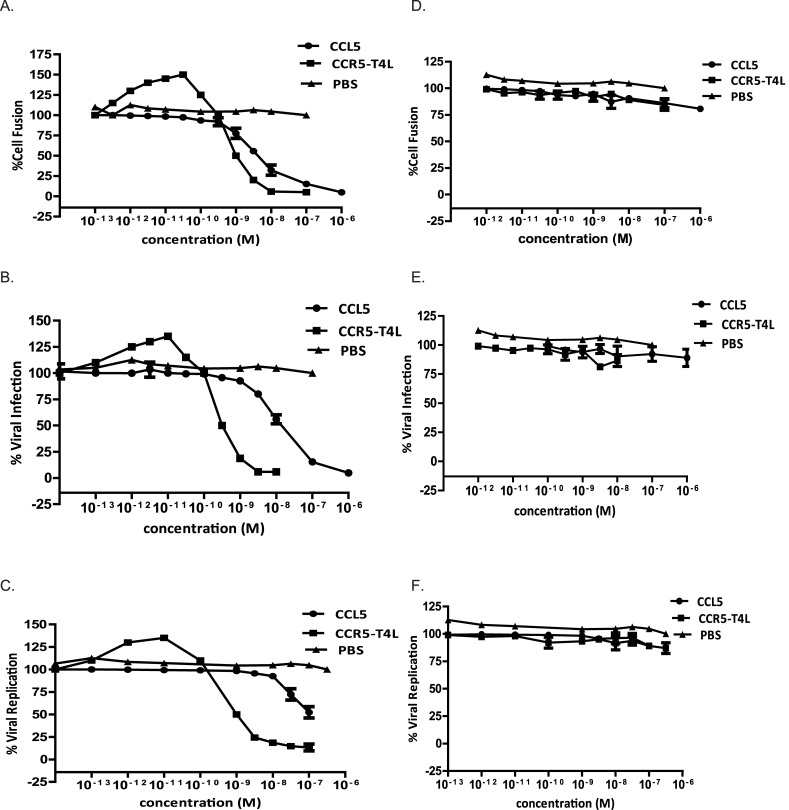
Antiviral activities of soluble recombinant CCR5-T4L against R5-tropic and X4-tropic virus. A and D) The inhibitory activities using cell-cell fusion assays. Effector HeLa cells co-expressing the R5 viral envelope BaL (A) or X4 (D) envelope LAV were pre-incubated for 30 min at 37°C with increasing concentrations of soluble recombinant CCR5-T4L plus 2 nM of soluble CD4. After washing with PBS, cell-cell fusion was performed. B and E) The inhibitory activities using single cycle viral infection assays in THP1 cells. Cells were pre-cultured overnight and infected with BaL or IIIB at 100 TCID_50_ in the presence or absence of soluble recombinant CCR5-T4L. Luciferase activity was analyzed using a luciferase kit 8 days post-infection. C and F) The inhibitory activities using replication competent viral assays with BaL strain virus- (C) and IIIB strain virus- (F) in THP1 cells. CCR5-tropic HIV-1 ADA was used as a positive control. THP1 cells (1×10^6^ cells per well) were pre-cultured overnight and infected with BaL or IIIB at 100 TCID_50_ in the presence or absence of soluble recombinant CCR5-T4L overnight. Supernatants were collected 7 days post-infection and tested for p24 antigen using ELISA. Data are the average from three independent experiments. Recombinant ligand CCL5 and PBS were used as controls.

As shown in Fig 3A, treatment of HeLa cells expressing R5 HIV Env BaL with low concentration (10^−12^ M level) of soluble recombinant CCR5-T4L protein plus 2 nM of soluble CD4 induced significant cell-cell fusion promotion. The highest fusion was achieved at 5×10^−10^ M. Further increases in recombinant protein concentration resulted in dose-dependent inhibition of cell fusion (Fig 3A). In the presence of CCL5 alone, only inhibition was observed. In contrast, treatment of HeLa cells expressing X4 HIV Env IIIB with same amount of protein had no effect on cell-cell fusion (Fig 3D). These data indicate that recombinant CCR5-T4L promotes R5 HIV Env-mediated cell-cell fusion, but not X4 HIV Env-mediated cell-cell fusion, at low concentrations and inhibits it at higher concentrations.

Similar results were obtained during further analyses of the inhibitory activities using single cycle viral infection assays in THP1 cells. As shown in Fig 3B, soluble recombinant CCR5-T4L protein promoted HIV infection at less than 10^−11^ M; at concentrations greater than 10^−10^ M, it inhibited viral infection. CCL5 alone only showed inhibitory effects. As expected, CCR5-T4L did not inhibit X4 envelope-pseudotyped virus infection (Fig 3E). These data demonstrated the specificity of soluble recombinant CCR5-T4L protein-mediated inhibition of CCR5.

A series of assays with replication-competent virus were also carried out. CCR5-tropic HIV BaL and CXCR4-tropic IIIB strains were used to infect THP1 cells using different concentrations of recombinant protein. Compared with the results of the single cycle viral infection assays, inhibition was less effective in viral replication. As shown in Fig 3C, treatment of cells with soluble recombinant CCR5-T4L protein either promoted or inhibited the R5 tropic virus depending on the CCR5-T4L concentration, but not X4 tropic virus replication (Fig 3F). As expected, CCR5-T4L did not inhibit X4 tropism viral strains. These data demonstrate the specificity of soluble recombinant CCR5-T4L protein-mediated inhibition of wild type CCR5 at higher concentration levels, which is approximately 2- to 6-fold more potent than CCL5-mediated inhibition in the corresponding viral strains.

### Antiviral potencies of soluble recombinant CCR5-T4L protein against R5-tropic HIV viruses in primary monocyte derived macrophage (MDM) cells and PBMCs from different donors

**Fig 4:**
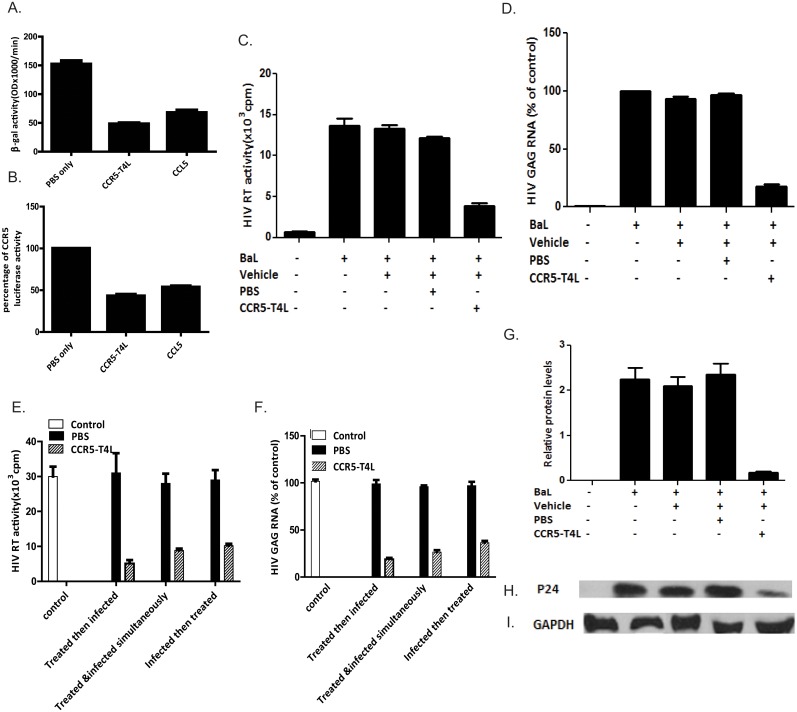
Soluble recombinant CCR5-T4L suppresses HIV infection in macrophages. A and B) Effect of soluble recombinant CCR5-T4L or CCL5 on macrophages using cell-cell fusion assays (A) and single round virus infection assays (B). Macrophages cultured for 7 days were stimulated for 24 h at 37°C with CCR5-T4L (1 μg/ml) or CCL5 (1 μg/ml) prior to HIV-1 Env-mediated cell-cell fusion or single round virus infection. C, D, G, H, and I) Effect of soluble recombinant CCR5-T4L on HIV BaL infection in macrophages. Macrophages cultured for 7 days were stimulated with CCR5-T4L (1 μg/ml) for 24 h at 37°C prior to HIV-1 Bal infection. Cultured supernatant was collected 8 days post-infection, and cells were collected 12 days post-infection. Supernatants were subjected to RT assays (C), total RNA was evaluated for HIV-1 *gag* expression using real-time PCR (D), and total protein extracted from cells was evaluated for HIV-1 p24 protein expression by western blot analyses (G and H) and GAPDH (I). Representative blots from three independent experiments are shown. Densitometry analyses of the blot were performed using Image J 1.44 software (NIH) and plotted into graphs (n = 3). E and F) CCR5-T4L suppresses HIV-1 replication in macrophages. Macrophages were cultured at 37°C for 24 h in conditioned media in the presence or absence of CCR5-T4L (1 μg/ml) prior to HIV-1 infection or simultaneously or 8 h post-infection. Supernatants were collected 8 days post-infection, and cells were collected 12 days post-infection. Supernatants were subjected to RT assays (E) and total RNA was evaluated for HIV-1 gag expression using real-time PCR (F). Data are expressed as RNA levels relative to control. The results represent the mean ± SD of three independent experiments.

To determine the potential antiviral effects of the soluble recombinant CCR5-T4L protein on primary human cells, we tested it on human monocyte-derived macrophages (MDM). Treatment of the effector HeLa cells, which expressed R5 HIV Env Bal, with soluble recombinant CCR5-T4L protein plus 2 nM of soluble CD4 inhibited cell-cell fusion (Fig 4A). In single cycle viral infection assays, CCR5-T4L protein showed a similar inhibitory effect on R5-tropic HIV virus in macrophage infection (Fig 4B). Further tests were performed using RT activity assays, *gag* RNA assays, and p24 protein assays to confirm the antiviral potencies of soluble recombinant CCR5-T4L protein. We stimulated macrophages with CCR5-T4L protein or PBS control before or after infection of the HIV BaL strain. As shown in Fig 4C and 4D, cells that were pretreated with CCR5-T4L before infection with HIV BaL showed a significant decrease in RT activity and *gag* gene expression. CCR5-T4L-mediated inhibition of HIV replication was also confirmed by diminished HIV p24 protein expression in macrophages stimulated with CCR5-T4L (Fig 4G–4I). We next examined suppression of HIV-1 replication in MDM using soluble recombinant CCR5-T4L protein. Cells stimulated with CCR5-T4L for 24 hours prior to HIV infection or treated and infected simultaneously showed lower levels of HIV replication compared to the 8-hour post-infection treated cells (Fig 4E and 4F).

Viral assays against PBMCs were performed to evaluate the R5-tropic antiviral potencies of soluble recombinant CCR5-T4L protein on natural human cells using inactivated cell-free HIV Bal and primary R5 isolates with different subtypes (92US657, 93MW959, or 92TH009). As shown in Table 1, soluble recombinant CCR5-T4L inhibited the replication of all R5 isolates, with 50% effective concentrations ranging from 58.6 to 147.6 nM. In contrast, the protein did not inhibit the replication of the X4 strain (HIV IIIB). The protein also did not affect the viability and proliferation of uninfected PBMCs at concentrations up to 10 μM (data not shown). Thus, soluble recombinant CCR5-T4L protein was extremely potent against R5 tropic virus even on natural human cells. It appears that the anti-HIV activity of soluble recombinant CCR5-T4L protein is often affected by host cells obtained from different donors. Therefore, the effects of the protein against two R5 strains (BaL and 92TH009) was examined in PBMCs from four different donors. In the absence of the protein, the p24 antigen levels in culture supernatants ranged from 24 to 63 ng/ml for the BaL strain and from 11 to 81 ng/ml for the 92TH009 strain on day 7 after virus infection (Table 2). These data indicate that the replication efficiency of R5 HIV differed considerably among donors. However, this difference in HIV replication was scarcely influenced by the anti-HIV activity of soluble recombinant CCR5-T4L protein. The protein inhibited R5 HIV virus replication, with EC_50_ ranging from 13.6 to 64.3 nM for the BaL strain and from 23.1 to 86.9 nM for the 92TH009 strain (Table 2).

**Table 1:** Anti-HIV activity of the soluble recombinant CCR5-T4L protein in PBMC assays.

Strain	Tropism	Cell type	EC_50_ (nM)
IIIB	X4	PBMC	>1000
BaL	R5	PBMC	58.6±6.2
92US657	R5	PBMC	147.6±6.8
93MW959	R5	PBMC	114.2±6.4
92TH009	R5	PBMC	86.9±5.7

**Table 2:** Anti-HIV activity of the soluble recombinant CCR5-T4L protein in PBMCs from four different donors.

Donor	Value in presence of BaL		Value in presence of 92TH009	
	EC_50_ (nM)	P24 (ng/ml)	EC_50_ (nM)	P24 (ng/ml)
1	58.6±6.2	24	86.9±5.7	28
2	13.6±3.5	63	23.1±2.7	11
3	64.3±7.1	48	58.3±3.6	81
4	42.6±5.3	34	73.2±4.5	59

### Soluble recombinant CCR5-T4L protein down-regulates surface CCR5 expression in human MDM

**Fig 5:**
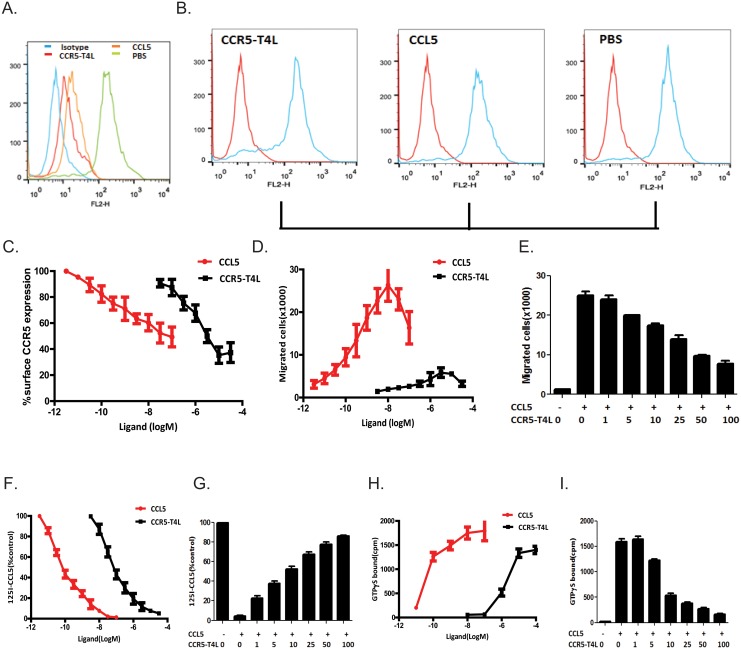
Recombinant CCR5-T4L down-regulates surface CCR5 expression in MDMs, and inhibits MDM migration and binding properties. A and B) Effect of soluble recombinant CCR5-T4L or CCL5 on surface CCR5 expression in MDMs. MDMs were treated with soluble recombinant CCR5-T4L (1 μg/ml), CCL5 (1 μg/ml), or PBS for 24 h at 37°C. Surface (A) and intracellular CCR5 (B) were analyzed using flow cytometry and the PE-conjugated monoclonal antibody (2D7). Recombinant CCL5 protein was used as a control. The cellular distribution of CCR5 receptors was analyzed by fixing and permeabilizing cells using BD Cytofix/Cytoperm buffer. Data shown are from one representative experiment that was independently repeated at least three times. C) Dose-dependent effects of CCR5-T4L or CCL5 on surface CCR5 expression in MDM. D and E) Dose-dependent effects on MDM migration by CCR5-T4L (D) or CCL5 (100 nM) plus different concentrations of CCR5-T4L protein (E). F and G) Dose-dependent effects on [125I]-CCR5 binding by CCR5-T4L (F) or CCL5 (100 nM) plus different concentrations of CCR5-T4L protein (G). H and I) Dose-dependent effects on CCL5-induced [^35^S]GTPγS binding to membranes from human macrophages cells treated with CCR5-T4L (H) or CCL5 (100 nM) plus different concentrations of CCR5-T4L protein (I). Data are the mean ± SD of triplicate cultures, which are representative of three experiments.

In order to analyze the effects of recombinant soluble CCR5-T4L protein on CCR5 surface expression in MDM, we used the CCR5 specific monoclonal antibody 3A9 to measure the cell surface expression of CCR5 by FACS. A substantial proportion of freshly-isolated human MDM expressed CCR5 on the cell surface, which was down-regulated by the chemokine ligand CCL5. Surface expression of CCR5 in MDM was also markedly down-regulated by prior treatment of cells with soluble recombinant CCR5-T4L protein (Fig 5A). As shown in Fig 5C, CCR5-T4L down-regulated surface CCR5 expression in a dose-dependent manner. The subcellular distribution of the receptor was further analyzed by fixing and permeabilizing cells using BD Cytofix/Cytoperm buffer (Fig 5B). These results indicate that soluble recombinant CCR5-T4L protein reduces CCR5 expression on the cell surface in human macrophages. CCR5 transcript levels in macrophages were assessed using real-time PCR. Treatment with 1 μM soluble recombinant CCR5-T4L protein for 1 hour at 37°C did not result in a decrease in the level of CCR5 transcription (data not shown).

### Soluble recombinant CCR5-T4L protein inhibits human macrophages migration induced by chemoattractant CCL5

To characterize the effects of purified soluble recombinant CCR5-T4L protein on human macrophages, we first measured macrophage migration in response to ligand CCL5 and soluble recombinant CCR5-T4L using *in vitro* chemotaxis assays. As shown in Fig 5D, macrophages showed a normal chemotactic response to the ligand CCL5. In contrast, soluble recombinant CCR5-T4L protein did not stimulate significantly chemotaxis in human macrophages at any of the concentrations tested. We further tested macrophage migration in response to ligand CCL5 in the presence of different amounts of the purified soluble recombinant CCR5-T4L protein. In the presence of soluble recombinant CCR5-T4L, the chemotactic response of macrophages to CCL5 was significantly inhibited (Fig 5E). Soluble recombinant CCR5-T4L protein exhibited a dose-dependent inhibition of CCL-5-induced human macrophage migration. In another set of experiments, human macrophages were pretreated with 50 μM recombinant CCR5-T4L protein for 30 min at 37°C, followed by two thorough washes with PBS, before testing for chemotaxis. The migratory responses of these cells to CCL5 were also markedly inhibited (data not shown). Our results suggest that recombinant CCR5-T4L protein may reduce CCR5 expression on the cell surface of human macrophages.

### [^125^I]CCL5 shows different binding properties to CCR5-T4L and wild type CCR5: the influence of G protein coupling

CCR5 binds to a number of ligands including CCL3 (MIP-1α), CCL4 (MIP-1β), and CCL5 (RANTES). To test whether soluble recombinant CCR5-T4L protein affects the biological functions of CCR5 in human macrophages, cells were pretreated with different amounts of recombinant CCR5-T4L protein, with or without the chemokine ligand CCL5, and tested for specific binding of [^125^I]CCL5. CCL5 was also used as positive control. We found that CCR5-T4L and [^125^I]CCL5 competed for the same binding site. Human macrophage membranes that were pretreated with CCL5 exhibited a higher binding affinity to [^125^I]CCL5 with an EC_50_ value of 0.046±0.004 nM compared to that of CCR5-T4L, which showed an EC_50_ of 0.888±0.068 nM (Fig 5F). The binding decreased approximately 15% when pretreated with 1 μM CCL5 and 100 μg/ml CCR5-T4L protein (Fig 5G).

Using [^35^S]GTPγS-based binding assays, we further investigated the response of recombinant CCR5-T4L protein to chemokine CCL5 stimulation in human MDM. We found that CCR5-T4L stimulation led to dose-dependent G-protein activation. The ligand CCL5 maintained the ability to activate G proteins (EC_50_ = 2.100±0.326 nM); this ability was reduced for recombinant CCR5-T4L protein (EC_50_ = 5.455±0.511 μM) (Fig 5H). As shown in Fig 5I, when 1 μM CCL5 ligand with or without increasing concentrations of the soluble recombinant CCR5-T4L protein induce [^35^S]GTPγS binding to human MDM in a concentration-dependent manner.

## Discussion

In this study, we demonstrated that insertion of the T4 lysozyme (T4L) into the C-terminus of the CC chemokine receptor 5 (CCR5) does not affect co-receptor function and enhances recombinant CCR5-T4L protein stability and solubilization in a bacterial expression system. The purified soluble recombinant CCR5-T4L protein showed significant antiviral potency in inhibiting R5 tropic HIV-1 virus infection only at higher concentrations in the THP1 cell line but enhanced viral infection at lower concentrations. Inhibition of CCR5 by the CCR5-T4L protein is approximately 2- to 6- fold higher than CCL5 ligand-mediated inhibition. We further determined the potential antiviral effects of this recombinant protein on primary human cells using inactivated cell-free HIV Bal and primary R5 isolates with different subtypes (92US657, 93MW959, and 92TH009). Our data indicate that soluble recombinant CCR5-T4L inhibited replication of all R5 isolates, with ED_50_ concentrations ranging from 58.6 to 147.6 nM. Furthermore, the activity of the recombinant CCR5-T4L protein against the two R5 strains (BaL and 92TH009) was examined in PBMCs from four different donors. We found that the replication efficiency of R5 HIV differed considerably among donors. However, this difference in HIV replication was scarcely influenced by the anti-HIV activity of soluble recombinant CCR5-T4L protein. CCR5-T4L inhibited R5 HIV virus replication, with EC_50_ values ranging from 13.6 to 64.3 nM for the BaL strain and from 23.1 to 86.9 nM for the 92TH009 strain. These results strongly suggest that soluble recombinant CCR5-T4L is extremely potent against R5 tropic virus on natural human cells.

To elucidate the mechanism of soluble recombinant CCR5-T4L, we examined the interaction between CCR5-T4L and WT-CCR5 in a transient expression system. Both CCR5-T4L and WT-CCR5 can be expressed on the cell surface. However, when both plasmids were co-transfected into target cells, cell surface expression of CCR5 was downregulated. CCR5-T4L reduced cell surface expression of WT-CCR5, resulting in dominant inhibition of R5 Env-mediated cell-cell fusion signal. This inhibition decreased as the amount of plasmid DNAs increased. Co-immunoprecipitation data showed that CCR5-T4L interacted with WT-CCR5. These results suggest that CCR5-T4L may interfere with functional expression of WT-CCR5 by heterodimerization with and cytoplasmic sequestration of WT-CCR5 protein, as is the case for CCR5Δ32 and CCR5-893(-) [35], and similar to heterodimerization among the chemokines CCR2, CCR5, and CXCR4. This interaction retains CCR5 in the endoplasmic reticulum, thereby resulting in reduced cell surface expression. Importantly, heteromers between the CCR2, CCR5, and CXCR4 can only bind a single chemokine with high affinity. This negative binding cooperativity between heteromerized chemokine receptors is not limited to the cognate chemokines of both receptors but extends to synthetic allosteric antagonists of these receptors [36]. Our data also showed that soluble recombinant CCR5-T4L protein slightly promoted HIV infection at low concentrations and inhibited infection at high concentrations. We propose that soluble CCR5-T4L may heterodimerize with WT-CCR5 on the cell surface at low concentrations, which increases HIV-1 co-receptor function. However, at high concentrations, it can directly compete with WT-CCR5 to interact with CD4 and CCR5 on target cells to block HIV-1 infection.

We described a bacterial system to express large amounts of CCR5 by fusion to T4L. Insertion of T4L into the C-terminus of wild type CCR5 yields up to 10 mg of purified soluble recombinant protein per 1 liter of bacteria culture. This is consistent with previous reports that showed T4L engineering improves the stability and solubilization of recombinant GPCR proteins [37, 38]. However, to date, no recombinant receptor-T4L fusion protein purified from *E*.*coli* has been reported to act as a HIV co-receptor. The purified CCR5-T4L variant showed high binding affinity for HIV R5 gp120-expressing cells and soluble CD4 in vitro; thus, it reduces the CD4- and CCR5-double positive target cell fused with Env-expressing cells. This evidence strongly suggests that the recombinant CCR5-T4L protein functions as a competitive inhibitor of wild type CCR5, demonstrating recombinant CCR5-T4L protein may be an effective HIV infection inhibitor. However, we have not yet tested whether recombinant proteins purified from membrane have the same anti-viral functions.

Our results further revealed that soluble recombinant CCR5-T4L inhibits the human macrophage chemotactic response to the chemoattractant CCL5. In contrast, the CCR5-T4L protein did not work as an efficient agonist of CCR5, although it induced receptor internalization in human monocytes.

In conclusion, we have shown the ability to express and purify milligrams of soluble functional recombinant CCR5-T4L protein using a pET20b expressing system. We found both inhibition and promotion of HIV-1 infection depending on the protein concentration. Our results demonstrated that at the nM level, CCR5-T4L inhibited R5 tropic HIV-1 virus infection and replication in cell lines and primary cells, including human macrophages and PBMCs from different donors. The efficiency of this recombinant protein may be considered useful for the future design and development of anti-HIV-1 therapeutic agents.
